# Use of Principal Components for Scaling Up Topographic Models to Map Soil Redistribution and Soil Organic Carbon

**DOI:** 10.3791/58189

**Published:** 2018-10-16

**Authors:** Xia Li, Greg W. McCarty

**Affiliations:** ^1^Department of Geographical Sciences, University of Maryland; ^2^Hydrology & Remote Sensing Laboratory, Agricultural Research Service, United States Department of Agriculture

**Keywords:** Environmental Sciences, Issue 140, Topography-based model, stepwise principal component regression, stepwise ordinary linear regression, digital elevation model, soil redistribution, soil organic carbon

## Abstract

Landscape topography is a critical factor affecting soil formation and plays an important role in determining soil properties on the earth surface, as it regulates the gravity-driven soil movement induced by runoff and tillage activities. The recent application of Light Detection and Ranging (LiDAR) data holds promise for generating high spatial resolution topographic metrics that can be used to investigate soil property variability. In this study, fifteen topographic metrics derived from LiDAR data were used to investigate topographic impacts on redistribution of soil and spatial distribution of soil organic carbon (SOC). Specifically, we explored the use of topographic principal components (TPCs) for characterizing topography metrics and stepwise principal component regression (SPCR) to develop topography-based soil erosion and SOC models at site and watershed scales. Performance of SPCR models was evaluated against stepwise ordinary least square regression (SOLSR) models. Results showed that SPCR models outperformed SOLSR models in predicting soil redistribution rates and SOC density at different spatial scales. Use of TPCs removes potential collinearity between individual input variables, and dimensionality reduction by principal component analysis (PCA) diminishes the risk of overfitting the prediction models. This study proposes a new approach for modeling soil redistribution across various spatial scales. For one application, access to private lands is often limited, and the need to extrapolate findings from representative study sites to larger settings that include private lands can be important.

**Figure:**
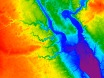


## Introduction

Soil redistribution (erosion and deposition) exerts significant impacts on soil organic carbon (SOC) stocks and dynamics. Increasing efforts have been devoted to investigating how SOC is detached, transported, and deposited over the landscape^1^,^2^,^3^. Carbon (C) sequestration and SOC distribution are influenced by gravity-driven soil movement induced by water erosion^4^,^5^,^6^. In cultivated fields, soil translocation by tillage is another important process contributing to C redistribution^7^,^8^,^9^. Tillage erosion causes a considerable net downslope movement of soil particles and leads to a within-field soil variation^10^. Both water and tillage erosion are significantly affected by landscape topography, which determines the locations of erosional and depositional sites^11^. Therefore, effective soil erosion regulation and C dynamic investigation in agricultural lands calls for a better understanding of topographic controls on soil erosion and movements.

Several studies have investigated the impacts of topography on soil redistribution and associated SOC dynamics^9^,^12^,^13^,^14^,^15^,^16^,^17^. Van der Perk* et al.*^12^ reported that topographic factors explained 43% of variability in soil redistribution. Rezaei and Gilkes^13^ found higher SOC in soils on a shady aspect, due to lower temperatures and less evaporation when compared to other aspects in rangelands. Topography may have more significant impacts on soil redistribution in agricultural lands with traditional tillage treatment than those with minimum tillage, due to the interactions between landforms and tillage practices^9^. However, these findings were primarily derived from field observations, which present difficulties in investigating soil properties at a broader spatial scale. There is a pressing need to develop new strategies to effectively understand spatial patterns of soil properties at watershed and regional scales.

The objective of this study is to develop efficient models to simulate soil redistribution and SOC distribution. Topography-based models using topographic metrics as predictors have been developed to quantify soil erosion and deposition processes. Compared with empirical- or process-based erosion models that employed discrete field samplings to simulate soil erosion^18^,^19^, topography-based models could be developed based on topographic information derived from digital elevation models (DEMs) with high resolutions. This approach allows for continuous soil property simulations at the watershed or regional scale. In the past several decades, accuracy of topographic information has substantially improved, with increasing availability of high resolution remotely sensed data. Although previous studies have employed topography-based models to simulate soil properties^12^,^20^,^21^,^22^, most of these investigations used a single topographic metric or single category of topographic metrics (local, non-local, or combined topographic metrics), which may not have sufficiently explored topographic impacts on soil microbial activity. Therefore, to gain a better understanding of topography controls on soil erosion and C dynamics, we examined a comprehensive set of topographic metrics including local, non-local, and combined topographic metrics and developed multi-variable topography-based models to simulate soil property dynamics. Applications of these models are expected to provide scientific support for better soil erosion control and agricultural land management.

Topographic metrics are generally categorized into one of three categories: a) local topographic metrics, b) non-local topographic metrics, or c) combined topographic metrics. Local topographic metrics refer to local features of one point on the land surface. Non-local topographic metrics refer to the relative locations of selected points. Combined topographic metrics integrate local and non-local topographic metrics. A set of topographic metrics affecting soil erosion and deposition were used in this study to investigate the topographic controls on soil movement and C stocks (**Table 1**). Specifically, we used four local topographic metrics [slope, profile curvature (P_Cur), plan curvature (Pl_Cur), general curvature (G_Cur)], seven non-local topographic metrics [flow accumulation (FA), topographic relief, positive openness (POP), upslope slope (UpSl), flow path length (FPL), downslope index (DI), catchment area (CA)], and three combined topographic metrics [topographic wetness index (TWI), stream power index (SPI), and slope length factor (LS)].

## Protocol

### 1. Topographic Analyses

1. Digital data preprocess
  1. Collect LiDAR data from the GeoTREE LiDAR mapping project website. Select "boundary type" and "region" to zoom into a specific area. Draw a polygon to download LiDAR tiles for the selected study area.
  2. Convert the raw LiDAR data to a LAS file using the geographic information system (GIS) mapping tool.
  3. Generate DEMs with a 3-m spatial resolution using inverse distance weighted interpolation.
  4. Filter the 3-m DEMs twice with a 3-kernel low pass filter to reduce noises associate with local variation.
2. Topographic metric generation
  1. To generate topographic metrics, first download the latest version of the System for Automated Geoscientific Analysis (SAGA)^23^. Click "Import Raster" in the Import/Export section to import the filtered 3-m DEMs into SAGA.
  2. Click the "Slope, Aspect, Curvature" module of SAGA with the default settings to generate the slope and curvature-related [profile curvature (P_Cur), plan curvature (Pl_Cur), and general curvature (G_Cur)] metrics using the filtered DEMs (Figure 1).
  3. Click the "Flow Accumulation (Top-Down)" module of SAGA and select "Deterministic infinity" as the method to generate flow accumulation (FA) metric using the filtered DEMs.
  4. Click the "SAGA Topographic Openness" module with the default settings to generate the positive openness (POP) metric using a filtered z-axis amplified image.
  5. Click the "LS- factor (Field Based)" module of SAGA with the default settings to generate the upslope slope (Upsl) and slope length factor (LS_FB) metrics using the filtered DEMs.
  6. Click the "Flow Path Length" module of SAGA with the default settings to generate the flow path length (FPL) metric using the filtered DEMs.
  7. Click the "Downslope Distance Gradient" module of SAGA with the default settings to generate the downslope index (DI) metric using the filtered DEMs.
  8. Click the "SAGA Wetness Index" module and select "absolute catchment area" as the Type of Area to generate the catchment area (CA) and topographic wetness index (TWI) metrics using the filtered DEMs.
  9. Click the "Stream Power Index" module of SAGA and select "pseudo specific catchment area" as the Area Conversion to generate the stream power index (SPI) metric using the filtered DEMs.
  10. Generate maximum elevation maps with multiple radiuses. Filter the maximum elevation maps twice through a 3-kernel low pass filter. Subtract the filtered 3-m DEM from the filtered maximum elevation maps to obtain a series of relief maps. Extract a series of relief variables to a number of locations.
  11. Perform principal component analysis (PCA) on the relief variables to convert the reliefs into topographic relief components. Select principal components that explain more than 90% variance of the relief dataset as the topographic relief metrics.

### 2. Field Data Collection

1. Field sampling
  1. Select a number of cropland field locations that can adequately represent the landscape characteristics of the study area and several representative small-scale cropland fields that can be intensively sampled. NOTE: The soil samples collected from the two cropland fields were used for model calibration. Soil samples collected from the entire study area were used for model validation.
  2. Upload all the sample location coordinates to a code-based geographic positioning system (GPS) and physically locate them in the fields.
  3. Collect 3 samples for each sampling location from the top 30 cm soil layer using a push probe (3.2 cm in diameter). ​NOTE: Soil samples from 30-50 cm layers were collected at sites where sediment deposition was expected. The volume of each sample was 241 cm^3^.
  4. Record geographic coordinate information of sampling locations using GPS.
  5. Weigh the soil samples after drying them at 90 °C for 48 h. Calculate soil density using information of total sample volumes at sampling locations and weights. Mix the three samples from the same location to get a composite soil sample.
2. Soil sample preparation
  1. Sieve the composite soil samples with a 2-mm screen.
  2. Grind a 10 g subsample of the sieved soil to a very fine powder with a roller mill.
3. Soil sample analyses
  1. Measure soil total carbon (C) content in roller milled samples through combustion on a CN elemental analyzer at a temperature of 1350 °C. Estimate calcium carbonate C content by analyzing the remaining C after baking soil organic matter at a temperature of 420 °C for 16 h in a furnace.
  2. Calculate SOC content (%) by subtracting calcium carbonate C content from total soil C content. Convert SOC content (%) to SOC density (kg m^-2^) using soil density.
  3. Put the bulk 2-mm sieved soil samples in Marinelli beakers and seal them. Measure ^137^Cs concentration of each sample through gamma-ray analysis using a spectroscopy system that receives inputs from three high purity coaxial germanium crystals (HpCN30% efficiency) into 8192-channel analyzers (see **Table of Materials**).
  4. Calibrate the system using an analytic mixed radionuclide standard^11^. Convert ^137^Cs concentration to ^137^Cs inventory using soil density.
  5. Calculate soil redistribution rate using ^137^Cs inventory by applying the Mass Balance Model II (MBMII) in a spreadsheet add-in program developed by Walling* et al.*^24^.

### 3. Topography-Based Model Development

1. Topographic principal component estimation
  1. Extract the topographic metrics for sampling locations in the entire study area and the small-scale cropland fields.
  2. Standardize the topographic metrics of the sampling locations in the entire study area by using mean and standard deviation. Estimate the topographic metric loadings in each component based on the standardized topographic metrics using PCA with statistical software package. Collect the topographic metric loadings in each topographic principal component (TPC) and select the top TPCs that explain 90% variance of all metrics.
  3. Standardize the topographic metrics of the sampling locations in the small-scale cropland fields. Calculate the top TPCs for each location by sum of the standardized topographic metrics weighted by the corresponding loadings from the sampling locations in WCW.
2. Model calibration
  1. Perform stepwise ordinary least square regression (SOLSR) to develop topography-based SOLSR_f_ models for SOC density and soil redistribution rates based on all topographic metrics at the small-scale cropland fields. Use Akaike information criterion (AIC) and leave-one-out cross-validation to select the optimal combination of topographic metrics for the best-fitted SOLSR_f_ models.
  2. Check the collinearity among the topographic variables using the variance inflation factor (VIF). Remove the variables with the largest VIF (VIF ≥ 7.5^25^), and check VIF again. Remove the variables until the VIFs of all variables are < 7.5. Perform SOLSR to develop topography-based SOLSR_r_ models for SOC density and soil redistribution rates based on topographic metrics that were removed high collinearity variables. Use the AIC and leave-one-out cross-validation to select the optimal combination for the best-fitted SOLSR_r_ models.
  3. Perform stepwise principal component regression (SPCR) to develop topography-based SPCR models for SOC density and soil redistribution rates based on the TPCs at the small-scale cropland fields. Use the AIC and leave-one-out cross-validation to select the optimal combination of TPCs for the best-fitted SPCR models.
  4. Calculate the adjusted coefficient of determination (R_adj_^2^), Nash-Sutcliffe efficiency (NSE), and ratio of the root mean square error to the standard deviation of measured data (RSR) to assess model efficiencies.
3. Model evaluation
  1. Estimate SOC density and soil redistribution rates in the entire study area by applying the estimated models.
  2. Validate the developed model by comparing prediction with measured dataset of SOC density and soil redistribution rates in the entire study area. Evaluate the model performances using R_adj_^2^, NSE, and RSR values.

## Representative Results

We used the Walnut Creek Watershed (WCW) as a testbed to assess feasibility of topography-based models in investigating soil redistribution and SOC dynamics. The watershed is in Boone and Story counties within the state of Iowa (41°55'-42°00'N; 93°32'-93°45'W) with an area of 5,130 ha (Figure 2). Croplands is the dominant land use type in the WCW, with a relatively flat terrain (mean 90 m, topographic relief 2.29 m). Chisel plowing, disking, and harrowing operations are the principal tillage practices in the crop fields^26^,^27^; however, tillage directions vary due to differences in management practices.

Four hundred and sixty crop field locations were randomly selected to derive topographic information in the WCW (Figure 2). 100 out of the 460 locations, including two 300 m transects (each have 9 sampling locations), were selected to conduct field samplings and for analysis of SOC and soil redistribution levels. In addition, two small-scale field sites with topographic landscape, soil types, and tillage practices similar to the WCW were selected for more intensive samplings. At each small-scale field site, a 25 × 25 m grid cell was created, and 230 sampling locations were located at grid nodes (Figure 3). Topographic metrics and soil property information were calculated for the 230 locations.

The topographic metrics in the WCW were generated following the above protocol. The WCW is characterized with low-to-moderate topography (elevation ranging from 260 to 325 m) with a relative low slope (ranging from 0 to 0.11 radian), upslope slope (0 to 0.09 m), and moderate curvatures (profile curvature: -0.009 to 0.009 m^-1^, plan curvature: -0.85 to 0.85 m^-1^, general curvature: -0.02 to 0.02 m^-1^). The vertical elevations of DEMs were enlarged 100 times to increase the distinguishability of the relatively low field-scale relief found in the WCW for creating the positive openness metrics (POP100). After conversion, the range of positive openness increased from 0.08 radians (POP: 1.51-1.59 radians) to 0.86 radians (POP100: 0.36-1.22 radians).

For the topographic relief, we generated seven relief maps with following radiuses: 7.5 m, 15 m, 30 m, 45 m, 60 m, 75 m, and 90 m. Two relief principal components were selected based on results of PCA on the seven relief variables. The first showed coarse resolution relief variation with relief_45m_ as the main variable. We defined this component as the large-scale relief (LsRe). The second component, which was highly correlated with relief_7.5m_ and presented fine resolution relief variation, was defined as the small-scale relief (SsRe).

Results of correlation analyses between topographic metrics and SOC density/soil redistribution are presented in **Table 2**. The TWI and LsRe showed the highest correlations with SOC density and soil redistribution rates, respectively. Spatial patterns of the two metrics are presented in Figure 4. Details of the TWI and LsRe can be better observed from the transect area. Both metrics showed high values in depressional area and low values in sloping and ridge areas. However, differences between the two metrics occurred in ditch areas, where the TWI exhibited extremely high values but the values of LsRe were not different from adjacent areas.

After generating the fifteen topographic metrics, we used PCA on these topographic variables over the 460 sampling sites in the WCW. The first seven topographic principal components (TPCs) that explained more than 90% variability of the whole topographic dataset were selected. Five TPCs that were final selected to build topography-based models are listed in **Table 3**. For the first principal component (TPC1), G_Cur showed the highest loading. Slope, TWI, Upsl, and LS_FB were the most important metrics in TPC2, with loadings larger than 0.35. In the TPC3, FA, SPI, and CA were important metrics, with loadings of 0.482, 0.460, and 0.400, respectively. FPL (-0.703) and Pl_Cur (0.485) were the most important in the TPC6. The main metrics with high loadings in the TPC7 were SsRe (0.597), DI (0.435), FPL (0.407), and Pl_Cur (0.383).

Collinearity of topographic variable was checked by examining VIF. Of the 15 metrics, slope, TWI, and G_Cur were removed due to the high VIFs. Based on soil redistribution rates and carbon density data from sites 1 and 2, SOLSR models were developed using all 15 metrics (SOLSR_f_) and the 12 metrics with collinear covariate removed (SOLSR_r_) (**Table 4**). Generally, over 70% and 65% of variability in SOC density and soil redistribution rates were explained by the SOLSR_f_ models, respectively. For the models with collinear covariate removed (SOLSR_r_), simulation efficiencies were slightly lower than SOLSR_f_ models (68% for SOC density and 63% for soil redistribution). NSEs were slightly lower and RSR were slightly higher in SOLSR_r _models than in SOLSR_f_ models.

For SPCR models, similar simulation efficiencies as SOLSR_r_ are observed in **Table 4**. However, fewer independent variables were selected in SPCR models (less than 5 variables) than the SOLSR_f_ and SOLSR_r_ models (more than 6 variables). TPCs 1, 2, 3, and 7 were selected as the independent variable combinations for the SOC model and TPCs 1, 2, 3, 6, and 7 were selected as the combination for the soil redistribution model.

We found that the SPCR models had the best predictions and the SOLSR_r_ models showed the poorest performances at the watershed scale. The coefficients of determination (r^2^) by comparing SOC density prediction to observation increased from: 1) 0.60 in SOLSR_f_ and 0.52 in SOLSR_r_ to 0.66 in SPCR, and 2) NSE increased from 0.21 in SOLSR_f_ and 0.16 in SOLSR_r_ to 0.59 in SPCR; while RSR reduced from 0.87 in SOLSR_f_ and 0.91 in SOLSR_r_ to 0.64 in SPCR. Soil redistribution rate prediction in SPCR accounted for 36% of the variability in the measured variable and was higher than the predictions by SOLSR_f_ (34%) and SOLSR_r_ (0.35%). A higher NSE and lower RSR in SPCR (NSE = 0.33, RSR = 0.82) compared to SOLSR_f_ (NSE = 0.31, RSR = 0.83) and SOLSR_r_ (NSE = 0.32, RSR = 0.82) also demonstrated a better performance in soil redistribution rate simulation by SPCR.

According to the model performance evaluations, SPCR models were selected to generate SOC density and soil redistribution rate maps at the watershed scale. The maps revealed consistent patterns between model simulations and field measurements (Figure 5). The high consistencies between simulations and observations were more evident along the transects. Both SOC density and soil redistribution rates showed high correlations with landscape topography. High values of SOC density can be found in footslope and depositional areas, where soil deposition occurred, while low values of SOC density were observed in sloping areas, where soil erosion took place.

**Figure:**
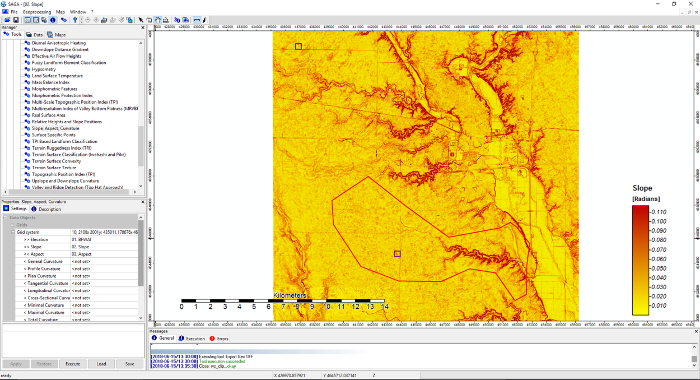

Figure 1**: The Slope, Aspect, Curvature module in the System for Automated Geoscientific Analysis (SAGA).** The polygons show the locations of study areas. Please click here to view a larger version of this figure.

**Figure:**
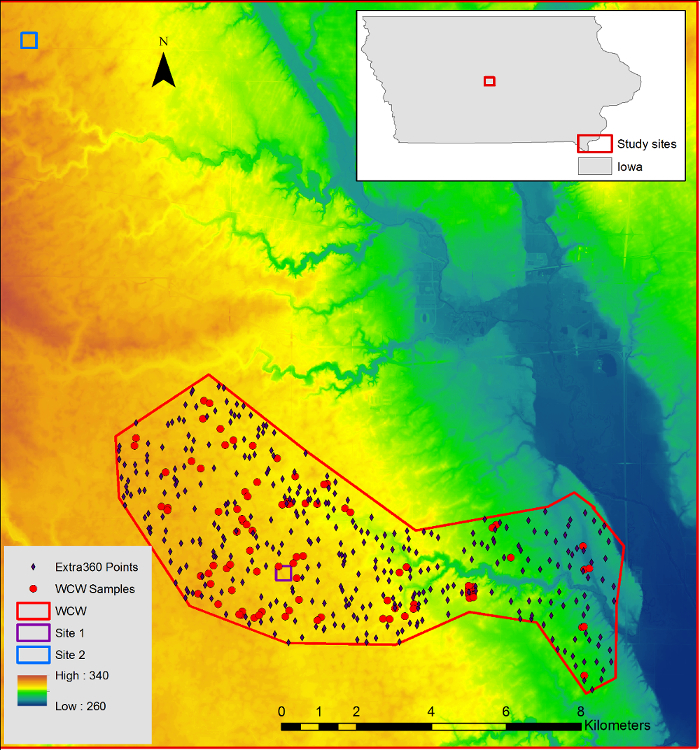

Figure 2**: Location of Walnut Creek Watershed and sampling sites in the watershed (Iowa).** This figure was adapted from previous work^17^. Please click here to view a larger version of this figure.

**Figure:**
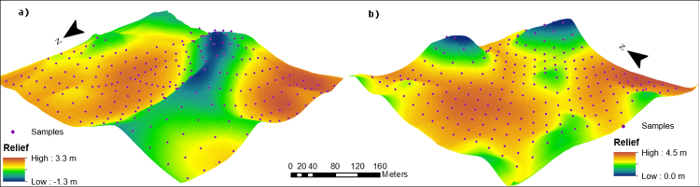

Figure 3**:** **Location of sampled sites a) 1 and b) 2 (z-axis 15x elevation).** This figure was adapted from previous work^17^. Please click here to view a larger version of this figure.

**Figure:**
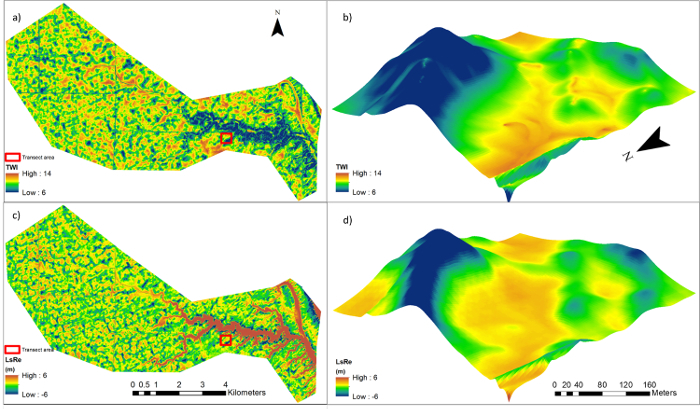

Figure 4**: Topographic metric maps.** (a) Topographic wetness index (TWI) and (b) large-scale topographic relief (LsRe) in the Walnut Creek Watershed and transect area (z-axis 15 x elevation). Please click here to view a larger version of this figure.

**Figure:**
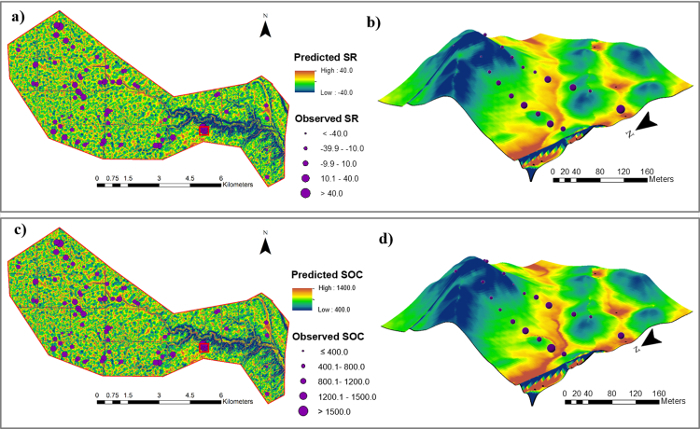

Figure 5**:Soil redistribution rate (t ha^-1^ year**^-1^**) maps and SOC density (kg m**^-2^**) maps. **Shown are soil redistribution maps (a) within the Walnut Creek Watershed and (b) along two transects. Shown are SOC density (kg m^-2^) maps (c) within the Walnut Creek Watershed and (d) along two transects using the stepwise principal component analysis models (z-axis 15x elevation). Please click here to view a larger version of this figure.

**Table:** 

Variables	Significance
Slope (radian)	Runoff velocity, soil water content^28^,^29^
Profile Curvature (m^-1^)	Flow acceleration, soil erosion, deposition rate^11^,^30^
Plan Curvature (m^-1^)	Flow convergence and divergence, soil water content^30^
General Curvature (m^-1^)	Runoff velocity , soil erosion, deposition^29^
Flow accumulation	Soil water content, runoff volume^20^
Topographic Relief (m)	Landscape drainage characteristics, runoff velocity and acceleration^21^,^31^
Positive Openness (radian)	Landscape drainage characteristics , soil water content^32^
Upslope Slope (m)	Runoff velocity^33^,^34^
Flow Path Length (m)	Sediment yield, erosion rate^35^
Downslope Index (radian)	Soil water content^36^
Catchment Area (m^2^)	Runoff velocity and volume^33^,^37^
Topographic Wetness Index	Soil moisture distribution^28^,^38^,^39^
Stream Power Index	Soil erosion, Convergence of flow^40^
Slope Length Factor	Flow convergence and divergence^28^,^40^

**Table 1:** **Significances of selected topographic metrics.**

**Table:** 

	Slope	P_Cur	Pl_Cur	G_Cur	FA	LsRe	SsRe	POP	Upsl	FPL	DI	CA	TWI	SPI	LS_FB
(radian)	(m^-1^)	(m^-1^)	(m^-1^)	(m)	(m)	(radian)	(m)	(m)	(°)	(m^2^)
SOC	-0.687	-0.159	-0.333	-0.288	0.165	0.698	-0.171	-0.451	-0.315	0.499	0.413	0.588	0.735	0.165	-0.453
***,†	**	***	***	***	***,†	***	***	***	***	***	***,†	***,‡	***	***
SR	-0.65	-0.205	-0.274	-0.282	0.156	0.687	-0.099	-0.427	-0.217	0.487	0.361	0.565	0.647	0.156	-0.438
***,†	***	***	***	**	***,‡	*	***	***	***	***	***,†	***,†	***	***
P_Cur, Pl_Cur, and G_Cur are profile curvature, plan curvature and general curvature, respectively; FA is flow accumulation; RePC1 and RePC2 are topographic relief component 1 and 2, respectively; POP100 is positive openness; Upsl is upslope slope; FPL is flow path length; DI is downslope index; CA is catchment area; TWI is topographic wetness index; and SPI is stream power index; and LS_FB is slope length factor (field based).
* P < 0.05, ** P < 0.005, *** P < 0.0001.
†Correlation coefficient >0.5, ‡Highest correlation coefficient for each soil property.

**Table 2: Spearman's rank correlation (n = 560) between selected topographic metrics and soil organic carbon (SOC) density and soil redistribution rates (SR).**

**Table:** 

	TPC1(25%)	TPC2(24%)	TPC3(14%)	TPC6(5%)	TPC7(4%)
Slope	0.062	0.475†	-0.035	-0.013	-0.183
P_Cur	-0.290	0.000	0.346	-0.070	-0.002
Pl_Cur	-0.283	0.107	-0.001	0.485†	0.383†
G_Cur	-0.353†	0.054	0.275	0.025	0.100
FA	0.297	-0.042	0.482†	0.179	0.131
LsRe	0.309	-0.193	-0.237	0.113	-0.116
SsRe	0.234	0.266	-0.118	0.084	0.597†
POP100	-0.330	0.092	0.258	-0.292	0.217
Upsl	0.187	0.419†	-0.143	-0.066	0.012
FPL	0.147	-0.168	-0.088	-0.703†	0.407†
DI	0.103	-0.220	-0.164	0.184	0.435†
CA	0.326	-0.128	0.4†	-0.160	-0.092
TWI	0.053	-0.465†	-0.067	0.185	-0.047
SPI	0.345	-0.014	0.46†	0.169	0.080
LS_FB	0.256	0.396†	0.050	0.011	-0.072
P_Cur, Pl_Cur, and G_Cur are profile curvature, plan curvature and general curvature, respectively; FA is flow accumulation; RePC1 and RePC2 are topographic relief component 1 and 2, respectively; POP100 is positive openness; Upsl is upslope slope; FPL is flow path length; DI is downslope index; CA is catchment area; TWI is topographic wetness index; and SPI is stream power index; and LS_FB is slope length factor (field based).
†Loadings> 0.35.

**Table 3: Variable loadings in the principal components (TPCs) calculated for topographic metrics (n = 460) in Walnut Creek Watershed.**

**Table:** 

	Model	R^2^_adj_	NSE	RSR
Stepwise principal component regression (SPCR)				
SOC	2.932-0.058TPC2-0.025TPC3+0.051TPC7+0.037TPC1†	0.68	0.69	0.56
SR	2.111+0.013TPC1+0.032TPC7-0.028TPC2-0.016TPC3-0.010TPC6	0.63	0.63	0.61
Stepwise ordinary least square regression (SOLSR_f_)				
SOC	2.755+0.021TWI+0.0004FPL-6.369G_Cur-5.580Slope+ 0.011LsRe+0.091DI+0.013SsRe+0.125LS_FB	0.7	0.71	0.55
SR	2.117+0.007LsRe-3.128Slope+0.109DI+0.010SsRe+0.0002FPL+ 0.801Upsl -4.442P_Cur	0.65	0.65	0.59
Stepwise ordinary least square regression with collinear covariate removed (SOLSR_r_)			
SOC	2.951+0.033LsRe-2.869Upsl+0.0006FPL+0.028SsRe+0.124DI-0.163LS_FB+0.007SPI-10.187P_Cur	0.68	0.68	0.56
SR	2.042+0.016LsRe-0.146LS_FB+0.118DI+0.017SsRe+0.0003FPL+ 0.070POP	0.63	0.64	0.6
† The order of TPCs is based on the stepwise selection steps				
R^2^_adj_ is adjusted coefficient of determination; NSE is Nash-Sutcliffe efficiency; RSR is ratio of the root mean square error (RMSE) to the standard deviation of measured data.
TPC represents topographic principal component. TWI is topographic wetness index; FPL is flow path length; P_Cur, Pl_Cur, and G_Cur are profile curvature, plan curvature and general curvature, respectively; LS_FB is slope length factor (field based); LsRe and SsRe are large-scale and small-scale topographic reliefs, respectively; DI is downslope index; and Upsl is upslope slope.

**Table 4: Models of soil organic carbon (SOC) density and soil redistribution rates (SR) for agricultural fields based on topographic metrics at sites 1 and 2.**

## Discussion

The SOLSR_f_ models had slightly better performances than the SPCR models in calibration at the field scale. However, some of the topographic metrics, such as SPI and CA (r > 0.80), are closely correlated with each other. The collinearity may add uncertainties to model predictions. Because of the multicollinearity among predictors, small changes in the input variables can significantly affect the model predictions^41^. Therefore, the SOLSR_f_ models tended to be unstable and showed low efficiencies in simulations of SOC density and soil redistribution rate at the watershed scale. The SPCR models substantially outperformed the SOLSR_f_ models in prediction of SOC distribution at the watershed scale. TPCs eliminate the multicollinearity by converting the fifteen topographic metrics into mutually independent (orthogonal) components. The conversion also uncovered underlying relationships among topographic metrics. As indicated by the high loadings (> 0.35) of topographic metrics to the components, the TPC1, TPC2, TPC3, TPC6, and TPC7 were associated with runoff velocity, soil water content, runoff volume, flow divergence, and flow acceleration, respectively. Spatial patterns of soil redistribution rates and SOC distribution were highly correlated with soil water content and runoff divergence in the WCW, which is consistent with the study of Fox and Papanicolaou^2^, which demonstrated that eroded soil from upland could be impacted by flow divergence in a low-relief agricultural watershed.

Moreover, fewer predictor variables in the SPCR models than the SOLSR_f_ and SOLSR_r_ models reduced the risk of over-fitting the prediction models^42^,^43^. There were more than six variables in all the SOLSR models, which may increase the difficulty of data interpretation and induce high variance in model simulations^41^,^44^,^45^. This may account for the lower prediction efficiencies in WCW by the SOLSR models than by the SPCR models.

Topography- based SPCR models have advantages in simulating soil redistribution and associated SOC dynamics. First, topographic information can be easily derived from DEMs. Recent increased accessibility of the high spatial resolution LiDAR data can help improve the accuracy of DEM-derived landscape topography and benefit investigations in regions with limited field observations. Second, using a set of topographic metrics and statistical analyses, the topography-based models can efficiently quantify soil redistribution and SOC distribution patterns. Third, the application of principal component can effectively reduce biases associated with multicollinearity of topographic metrics and increase the stability of the stepwise regression models when applied to multiple spatial scales.

However, the SPCA models may be limited by variables during model development. Although application of the LiDAR data increased in ecological studies, the methods to derive useful topographic information have not yet been fully explored. In this study, the TWI and LsRe showed the highest correlations with SOC density and soil redistribution rates, respectively. However, additional topographic variables that are not considered may be equally or more important in explaining soil erosion and C dynamics. Additionally, other factors such as management practices, which may cause soil erosion variability, were not included in this study. For example, when tillage was parallel to the direction of maximum slope, soil erosion may double relative to the erosion in slantwise tillage turning soil upslope^46^. Therefore, different tillage practices may also be a reason for the reduced prediction efficiencies of the SPCR models.

The study is based on the paper published in Catena^17^. Instead of a mechanistic-based analysis of topographic influences on soil movement and soil properties as performed in the Catena paper, here we focused on the methods for quantifying topographic metrics and developing topography-based models. We discussed the feasibility and advantages of using topography-based models in studies of the spatial structure of soil properties. Meanwhile, we improved our models by updating algorithms of slope length factor and flow accumulation. The scale of slope length factor measurement was limited to field's area. Additionally, the deterministic infinity algorithm was used for flow accumulation generation. Compared with the method reported in Li* et al.*^17^ which generated flow accumulation with a deterministic eight-node algorithm, the infinity algorithm adopted in this study reduces loops in the flow direction angles and proved to be a better algorithm for low relief areas^47^.

In conclusion, our results demonstrate the feasibility of topography-based SPCR models in simulating SOC distribution and soil redistribution patterns in agriculture fields. As a cost-effective method to estimate SOC stocks and soil redistribution rates, it is applicable to sites with limited observational data and private lands lacking public access. In future studies, the prediction models could be improved with further refinement and availability of LiDAR data and inclusion of additional topographic metrics. The large-scale soil property maps that were developed based on the models will lead to further understanding of the mechanisms underlying the topographic impacts on soil movement in agricultural landscapes and the fate of SOC at the watershed and regional scales.

## Disclosures

The authors have nothing to disclose. 
